# Combination of three cytotoxic agents in small-cell lung cancer

**DOI:** 10.1007/s00280-012-2022-8

**Published:** 2012-11-18

**Authors:** G. P. Stathopoulos, D. Trafalis, J. Dimitroulis, Ch. Kosmas, J. Stathopoulos, D. Tsavdaridis

**Affiliations:** 1 First Oncology Clinic, Errikos Dunant Hospital, Semitelou 2A, 115 28 Athens, Greece; 2 6th Pulmonary Clinic, Hospital for Thoracic Diseases, Athens, Greece; 3 Oncology Clinic, Anticancer Hospital, Piraeus, Greece; 4 Oncology Clinic, Anticancer Hospital, Thessaloníki, Greece

**Keywords:** Small-cell lung cancer, Three-drug combination treatment, Three drugs small-cell lung cancer

## Abstract

**Purpose:**

The established treatment for small-cell lung cancer has been a cisplatin–etoposide combination, as the most effective chemotherapy regimen. Paclitaxel has also been used in combination with cisplatin and etoposide but this has been unacceptable due to the toxicity. This toxicity could be attributed to the three consequent days of treatment with etoposide plus the doses of each of the three drugs. Our objectives were to determine an equal or longer survival and lower toxicity by administering all 3 drugs with low dosage on day one, compared to the established guideline of 3-day administration.

**Methods:**

We tested the aforementioned three-drug combination and avoided the toxicity in the majority of patients by administering all 3 drugs on day one. Fifty-one patients (50 evaluable) were recruited from 4 oncology clinics. All patients had histologically or cytologically confirmed small-cell lung cancer with limited and extensive disease in 40 and 60 % of the patients, respectively. The treatment was: cisplatin 75 mg/m^2^, etoposide 120 mg/m^2^ (maximum 200 mg), and paclitaxel 135 mg/m^2^. The agents were administered on day one and repeated every 3 weeks for 6 cycles.

**Results:**

The median survival was 15 months (95 % CI 13.6–16.4) (mean 16 months). Forty-five (90 %) patients achieved a response: 20 (40 %) patients, a complete response and 25 (50 %), a partial response. Adverse reactions included grade 3 and 4 neutropenia in 12 and 2 % of the patients, respectively. Other side effects were of very low toxicity.

**Conclusion:**

The 1-day, three-agent (cisplatin–etoposide–paclitaxel) treatment of small-cell lung cancer is beneficial with respect to response rate and survival, and the toxicity is low and well-tolerated.

## Introduction

According to the chemotherapy guidelines, chemotherapy treatment of small-cell lung cancer (SCLC) has, for many years now, been the combination of cisplatin and etoposide [1]. Carboplatin has been substituted for cisplatin in order to avoid the nephrotoxicity of the latter, but no statistically significant difference was determined with respect to survival [2]. Many other cytotoxic combinations have been tested, without achieving better results [3–9]. SCLC is not an uncommon malignancy and it is detected in nearly 20 % of patients with lung carcinomas [10]. SCLC is a malignancy sensitive to chemotherapy and radiation therapy as the great majority of treated patients achieve complete and partial responses [11, 12]. Despite the initial responses in a high percentage of patients, disease recurrence is very common in about 85–90 %. The cytotoxic agents used, apart from cisplatin and etoposide, have been alkylating agents, anthracyclines, vinca alkaloids, taxanes, and camptothecins [3, 7, 13]. The 5-year survival rate has been reported to be relevant to only a small number of patients. Etoposide became an eligible agent for SCLC treatment as it has been administered in trials as monotherapy, given for several consecutive days and showing an 81–87 % response rate and a median survival of 7.1–9.4 months [11]. Chemotherapy has been shown to be more effective in limited disease as the median, and overall survival has been statistically significantly longer than in extensive disease [14]. Similar studies have been performed with approximately the same results [15, 16].

Future studies to find a substitute for the standard treatment of cisplatin–etoposide are probably needed. One may consider that the cisplatin–etoposide combination comes with two problems in clinical practice: the first is the toxicity which is quite high, particularly with high dosage cisplatin [7, 16], and second, the 3-day duration of etoposide administration.

In the present trial, the three agents, cisplatin, etoposide, and paclitaxel, which are already considered to be the most effective, are all given on 1 day every 3 weeks. The objectives of the present study were to determine an equal or longer survival and lower toxicity compared to the established guideline of 3-day drug administration.

## Patients and methods

### Eligibility criteria

The eligibility criteria included patients with limited and extensive small-cell lung cancer disease, histologically or cytologically confirmed, a performance status (PS) of ≤2 (ECOG scale) and a life expectancy of at least 12 weeks. Patients were required to have adequate bone marrow function (absolute neutrophil count ≥1.5 × 10^9^/l, platelet count ≥100 × 10^9^/l and hemoglobin ≥100 g/l), adequate liver function (total bilirubin ≤1.5 times the upper normal limit, AST and/or ALT ≤3 times the upper normal limit), and a creatinine clearance rate of ≥60 ml/min. Patients with asymptomatic brain metastases were eligible. Patients with cardiac arrhythmias, heart failure, AV block or acute myocardial infarction within 4 months before study entry, as well as those with concurrent or previous malignancies (except adequately treated squamous-cell carcinoma of the skin) were excluded. The lower age limit for enrollment was 18 years. All patients gave their written informed consent and the protocol was approved by the hospitals and the local ethics regulatory bodies.

### Study design and sample size

This study was designed as a multicenter Phase II trial with four participating hospitals. The study was powered at 80 % to determine the response rate and survival. The sample size was initially planned to include 20 patients with an increased number of patients if 5 %, with regard to median survival, and response rate was not reached. If there had been no responses, the treatment would have been stopped. The evaluation was performed centrally and stratified by three prognostic variables: disease stage (limited vs. extensive disease) a PS of 0–2 and the investigational site.

### Treatment plan

All patients were designated to receive six cycles of the three anticancer agents: cisplatin (CDDP), etoposide and paclitaxel (PCT). The doses of all agents were lower than the dosage which would have been given if only two of the agents had been combined: CDDP 75 mg/m^2^ for 2 h plus 1½ l of normal saline hydration, etoposide 120 mg/m^2^ (the usual daily dose) and not higher than 200 mg administered for 30 min and PCT 135 mg/m^2^ for a 3-h infusion. The dose reduction of all 3 agents had to be done because the 3-drug administration at a higher dose would have increased the toxicity. The drugs were administered the first day only, and the courses were repeated every 21 days (3 weeks). Treatment was performed at an outpatient clinic. Patients who responded to the treatment continued up to the end of six cycles. Hemopoietic growth factor was not applied prophylactically, but only in cases of grade 3 and 4 neutropenia.

### Baseline and treatment assessment and evaluation

Before study entry, all patients underwent the following evaluations: medical history, physical examination, tumor measurement or evaluation, ECOG performance status, ECG, full blood count, liver and renal function test and urinolysis. Staging was determined by chest and abdominal computed tomography, bone scan and occasionally magnetic resonance imaging. Blood count, blood urea, and serum creatinine were measured before each treatment administration and 7 days after each course. Radiologic tests were conducted after the current course of treatment if the clinical signs were indicative of disease progression, or at the end of six courses. For the assessment of response, we used imaging-based evaluation. A complete response (CR) was defined as the disappearance of all measurable disease confirmed at 4 weeks at the earliest; partial response (PR), a 30 % decrease in tumor burden, also confirmed at 4 weeks at the earliest. In stable disease (SD), neither PR nor progressive disease (PD) criteria were met; PD, a 20 % increase in tumor burden and no CR, PR or SD before increased disease. Response data were based on the response evaluation criteria in solid tumors (RECIST) [17]. A two-step deterioration in performance status, a >10 % loss in pre-treatment weight or increasing symptoms did not, by themselves, constitute progression of the disease; however, the appearance of these complaints was followed by a new evaluation of the extent of the disease. All responses had to be maintained for at least 4 weeks and to be confirmed by two independent radiologists and three experienced oncologists.

### Statistical design

The study was designed as a group sequential clinical trial and an intent-to-treat analysis. An interim analysis based on the O’Brien/Fleming boundary values was performed when 50 % of the end points had been reached. Stratification factors comprised limited and extensive disease. Pearson’s x^2^ test (or Fisher’s exact test, when appropriate) was used for the comparisons of categorical variables. Time-to-event analysis was performed and survival distribution was estimated by the Kaplan–Meier curve. All reported *p* values are two-sided. A *p* < 0.05 was considered significant. The end points were median and overall survival, response rate, and toxicity.

## Results

From January 2008 till July 2011, 51 patients were enrolled in the study. One patient was excluded, having stopped treatment after the first course. Fifty patients received chemotherapy and nearly all completed the planned courses and were evaluable. The patients’ demographic and disease characteristics at baseline are shown in Table 1. There were 39 males and 11 females, 20 patients with limited disease and 30 with extensive. The median age was 64 years (range 45–83 years).

**Table 1:** Patients’ demographics and disease characteristics at baseline

	*n*	%
Recruited	51	100
Evaluated	50	98
Gender		
Male	39	78
Female	11	22
Age		
Median	64	
Range	45–83	
Performance status		
0	20	40
1	25	50
2	5	10
Histology		
SCLC	50	100
Disease stage		
Limited disease	20	40
Extensive disease	30	60

### Compliance with treatment

The total number of chemotherapy cycles was 278 and the median number of cycles was 5. The median interval for each patient was 21 days. No delay of treatment was necessary apart from 2 patients who had myelotoxicity, in which cases the delay was 1 week. There was no reduction in treatment. Growth factor was given to only 2 patients. Forty-six (92 %) of the patients completed the treatment.

### Response to treatment

Responses were analyzed on an intent-to-treat basis. Responses were observed in 45 patients (90 %), 20 with a complete response (40 %) and 25 with a partial response (50 %), stable disease in 3 patients (6 %) and disease progression in 2 patients (4 %), as shown in Table 2. Complete responses were observed in patients with limited and extensive disease.

**Table 2:** Response to cisplatin–paclitaxel–etoposide treatment

Response	*n* (%)
CR	20 (40)
PR	25 (50)
SD	3 (6)
PD	2 (4)
Total	50 (100)

### Survival

**Fig. 1:**
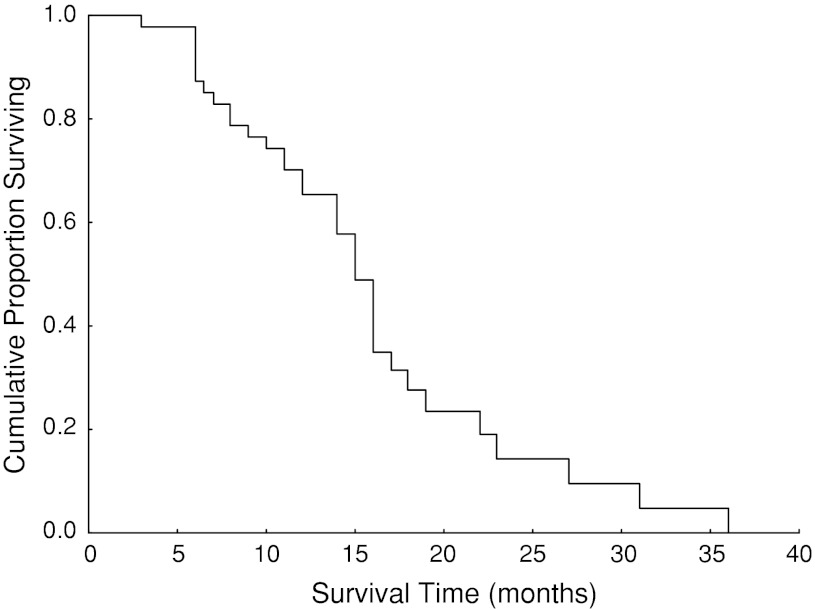
Kaplan–Meier survival curve. Median survival time 15 months, 95 % confidence interval 13.6–16.4

The median survival was 15 months (95 % confidence interval 13.6–16.4) (mean 16 months). All of the patients with limited and extensive disease were included. It is important to note that in the survival of over a year, 60 % of the patients had had extensive disease. Fourteen/20 (70 %) patients with limited disease had a survival rate of 15 months or longer, whereas 6/30 (20 %) patients with extensive disease had a survival rate of 15 months or longer. The Kaplan–Meier survival curve is shown in Fig. 1.

### Toxicity

Serious adverse reactions were not common. Alopecia occurred in 68 % of the patients. Myelotoxicity, and in particular neutropenia of different grades, was the most common. Serious grade 3 and 4 neutropenia occurred in 12 and 2 % of the patients, respectively; for grade 1 and 2, it was 14 and 28 %, respectively, which did not create a need for treatment delay. Grade 3 thrombocytopenia was experienced by 6 % of the patients and grades 1 and 2, 18 %; there was no grade 4 thrombocytopenia. Neuropathy in total was experienced by 20 % of the patients. The data related to adverse reactions are shown in Table 3.

**Table 3:** Toxicity

Adverse reactions	Grade 1 *n* (%)	Grade 2 *n* (%)	Grade 3 *n* (%)	Grade 4 *n* (%)	Total *n* (%)
Neutropenia	7 (14)	14 (28)	6 (12)	1 (2)	28 (56)
Thrombocytopenia	8 (16)	1 (2)	3 (6)	–	12 (24)
Anemia	3 (6)	5 (10)	2 (4)	–	10 (20)
Neuropathy	3 (6)	6 (12)	1 (2)	–	10 (20)
Mucositis	1 (2)	1 (2)	–	–	2 (4)
Nephrotoxicity	1 (2)	–	–	–	1 (2)
Asthenia	4 (8)	4 (8)	–	–	8 (16)
Anorexia	2 (4)	–	–	–	2 (4)
Nausea/vomiting	1 (2)	2 (4)	–	–	3 (6)
Allergy	1 (2)	–	–	–	1 (2)
Alopecia	–	–	34 (68)	–	34 (68)
Cardiotoxicity	–	–	–	–	–

### Second-line treatment

Patients with recurrence after a complete response or disease progression underwent second-line chemotherapy with irinotecan monotherapy or in combination with carboplatin (or cisplatin). Twenty-three patients were treated: 5 had a complete response; 6 had a partial response; 4 had stable disease; and 8 had disease progression.

All patients with limited disease underwent radiation therapy at the primary site (the dose was 4,000 cGy). Brain irradiation (the dose was 3,000 cGy) was performed when metastases appeared.

## Discussion

In trying to improve the results of chemotherapy in SCLC, quite a number of studies have been done. The combination of cisplatin with etoposide has remained in the chemotherapy guidelines. Cyclophosphamide with doxorubicin and vincristine showed no difference in effectiveness in SCLC [15]. This three-drug combination only increased the toxicity. The combination of cisplatin–etoposide–ifosfamide did not improve the effectiveness but the toxicity was increased [9, 18]. The fact that there is a need to increase effectiveness and the survival rate, as well as to reduce tumor recurrence, is a current reality for oncologists. Although there have been numerous attempts to improve the treatment, the optimal duration of chemotherapy in order to achieve an amelioration has not been defined. Evidence available from reported randomized trials with respect to maintenance chemotherapy showed no prolongation of survival [19–22]. Another attempt to improve the survival rate without reducing the quality of life in SCLC was made by increasing the dose-intensity of chemotherapy with granulocyte colony-stimulating factor support [23]. Another trial used the dose-intensity of a four-drug chemotherapy regimen with or without recombinant human granulocyte–macrophage colony-stimulating factor in extensive-stage small-cell lung cancer [24]. The efforts of the aforementioned trials did not become part of clinical practice because no further effectiveness was reported. In our trial, three agents were used in combination. With respect to the chemotherapy guidelines, 2 of the 3 drugs were cisplatin and etoposide and the third agent was paclitaxel. These 3 drugs have been used in the other trials. One of these was in a randomized trial, while in another study, the 3 drugs were administered in a Phase I study [25, 26]. The main outcome of these trials was toxicity due to the drug dosage and to the three-day treatment of etoposide. The comparison of cisplatin–etoposide versus cisplatin–paclitaxel has also been tested without producing different results in effectiveness and survival [27].

In the present Phase II trial, the objective was to increase the survival rate and reduce the toxicity, with the use of the three agents cisplatin, etoposide, and paclitaxel. Whenever three agents are combined in cancer clinical practice, one has to be wary of the possible adverse reactions. By reducing the doses of the three drugs and administering all three on day one, and repeating the treatment after 3 weeks, the toxicity was well**-**tolerated, the effectiveness was high, and the survival rate was one of longest reported in SCLC treatment. It is also important to take into account, the better quality of life of the patients in receiving the drug administration once every 3 weeks.

## Conclusion

In the present trial, the three cytotoxic agents, cisplatin, etoposide, and paclitaxel, were administered on day one, every 3 weeks, thus avoiding the three-day treatment and reducing the established drug dosage. The effectiveness and survival were quite good and the toxicity very low and very well-tolerated.

## References

[1] Loehrer P, Einhorn L, Greco FA (1988). Cisplatin plus etoposide in small cell lung cancer. Semin Oncol.

[2] Skarlos DV, Samantas E, Kosmidis P (1994). Randomised comparison of etoposide-cisplatin vs etoposide-carboplatin and irradiation in small-cell lung cancer. Ann Oncol.

[3] Jett JR, Everson L, Therneau TM (1990). Treatment of limited stage small-cell lung cancer with cyclophosphamide, doxorubicin and vincristine with or without etoposide: a randomised trial of the North Central Cancer Treatment Group. J Clin Oncol.

[4] Ettinger DS, Finkelstein DM, Ritch PS (2002). Study of either ifosfamide or teniposide compared to a standard chemotherapy of extensive disease small-cell lung cancer: an Eastern Cooperative Oncology Group randomised study (E1588). Lung Cancer.

[5] Lowenbraun S, Birch R, Buchanan R (1984). Combination chemotherapy in small cell lung cancer carcinoma: a randomised study of two intensive regimens. Cancer.

[6] Johnson DH, Einhorn LH, Birch R (1987). A randomised comparison of high-dose versus conventional-dose cyclophosphamide, doxorubicin and vincristine for extensive-stage small cell lung cancer: a Phase III trial of the Southeastern Cancer Study Group. J Clin Oncol.

[7] Fukuoka M, Furuse K, Saijo N (1991). Randomised trial of cyclophosphamide, doxorubicin and vincristine versus cisplatin and etoposide versus alternation of these regimens in small-cell lung cancer. J Natl Cancer Inst.

[8] Ettinger DS, Finkelstein DM, Sarma RP (1995). Phase II study of paclitaxel in patients with extensive-disease small-cell lung cancer: an Eastern Cooperative Oncology Group study. J Clin Oncol.

[9] Loehrer PJ, Rynard S, Ausari R (1992). Etoposide ifosfamide and cisplatin in extensive small-cell lung cancer. Cancer.

[10] Jemal A, Tiwari RC, Murray T (2004). Cancer statistics 2004. CA Cancer J Clin.

[11] Clark PI, Slevin ML, Joel SP (1994). A randomised trial of two etoposide schedules in small-cell lung cancer; the influence of pharmacokinetics on efficacy and toxicity. J Clin Oncol.

[12] Moore TD, Korn EL (1992). Phase II trial design considerations for small-cell lung cancer. J Natl Cancer Inst.

[13] Giaccone G, Ferrari P, Donadio M (1987). Reinduction chemotherapy in small-cell lung cancer. Eur J Cancer Clin Oncol.

[14] Sundstrom S, Bremnes RM, Kaasa S (2002). Cisplatin and etoposide regimen is superior to cyclophosphamide, epirubicin and vincristine regimen in small-cell lung cancer. Results from a randomised Phase III trial with five years’ follow up. J Clin Oncol.

[15] Roth BJ, Johnson DH, Einhorn LH (1992). Randomized study of cyclophosphamide, doxorubicin and vincristine versus etoposide and cisplatin versus alternation of these two regimens in extensive small-cell lung cancer: a Phase III trial of the Southeastern Cancer Study Group. J Clin Oncol.

[16] Einhorn LH, Crawford J, Birch R (1988). Cisplatin plus etoposide consolidation following cyclopphosphamide, doxorubicin and vincristine in limited small-cell lung cancer. J Clin Oncol.

[17] Therasse P, Arbuck SG, Eisenhauer EA (2000). New guidelines to evaluate the response to treatment in solid tumors. J Natl Cancer Inst.

[18] Loeher PJ, Ansari R, Gonin R (1995). Cisplatin plus etoposide with or without ifosfamide in extensive small cell lung cancer: a Hoosier Oncology Group Study. J Clin Oncol.

[19] Spiro SG, Souhami RL, Geddes DM (1989). Duration of chemotherapy in small cell lung cancer: a Cancer Research Campaign trial. Br J Cancer.

[20] Bleehen NM, Girling DJ, Machin D (1993). A randomised trial of three to six courses of etoposide, cyclophosphamide, methotrexate and vincristine or six courses of etoposide and ifosfamide in small cell lung cancer (SCLC) I: survival and prognostic factors. Medical Research Council Lung Cancer Working Party. Br J Cancer.

[21] Giaccone G, Dalesio O, McVie GJ (1993). Maintenance chemotherapy in small-cell lung cancer: long term results of a randomised trial. European Organisation for Research and Treatment of Cancer, Lung Cancer Cooperative Group. J Clin Oncol.

[22] Sculier JP, Paesmans M, Bureau G (1996). Randomized trial comparing induction chemotherapy versus induction chemotherapy followed by maintenance chemotherapy in small-cell lung cancer. European Lung Cancer Working Party. J Clin Oncol.

[23] Thatcher N, Girling DJ, Hopwood P (2000). Improving survival without reducing quality of life in small-cell lung cancer patients by increasing the dose-intensity or chemotherapy with granulocyte colony-stimulating factor support: results of a British Medical Research Council. Multicenter randomized trial. Medical Research Council Lung Cancer Working Party. J Clin Oncol.

[24] Pujol JL, Douillard JY, Rivière A (1997). Dose-intensity of a four-drug chemotherapy regimen with or without recombinant human granulocyte-macrophage colony-stimulating factor 1997 in extensive-stage small-cell lung cancer: a multicenter randomized Phase III study. J Clin Oncol.

[25] Mavroudis D, Papadakis E, Veslemes V (2001). A multicenter randomized clinical trial comparing paclitaxel-cisplatin-etoposide versus cisplatin-etoposide as first line treatment in patients with small cell lung cancer. Ann Oncol.

[26] Bunn PA, Kelly K (1997). A Phase I study of cisplatin, etoposide and paclitaxel in small-cell lung cancer. Semin Oncol.

[27] Dimitroulis J, Rapti A, Stathopoulos GP (2008). Comparison of cisplatin-paclitaxel-etoposide combination versus cisplatin-etoposide in patients with small-cell lung cancer. A Phase III study. Oncol Rep.

